# Deciphering the Potential Causal and Prognostic Relationships Between Gut Microbiota and Brain Tumors: Insights from Genetics Analysis and Machine Learning

**DOI:** 10.1002/EXP.20240087

**Published:** 2025-05-01

**Authors:** Changwu Wu, Fushu Luo, Yongye Zhu, Chunbo Liu, Zheng Chen, Xiangyu Wang, Jun Tan, Qing Liu

**Affiliations:** ^1^ Department of Neurosurgery Xiangya Hospital Central South University Changsha Hunan China; ^2^ National Clinical Research Center for Geriatric Disorders Xiangya Hospital Central South University Changsha Hunan China; ^3^ Clinical Research Center for Skull Base Surgery and Neuro‐Oncology in Hunan Province Changsha Hunan China; ^4^ Institute of Anatomy University of Leipzig Leipzig Germany

**Keywords:** glioma, gut microbiota, machine learning, mendelian randomization, meningioma, prognosis

## Abstract

The concept of the microbiota‐gut‐brain axis has witnessed significant advancements, and observational studies revealed dysbiosis in the gut microbiota of patients with brain tumors. The causal relationship between gut microbiota and brain tumors and the potential prognostic value of microbiota are still unclear. Based on multiple Mendelian randomization analyses, this study confirms the causal effects of four gut microbes on meningioma, seven gut microbes on pituitary tumor, and eight gut microbes on glioma. Based on the Sherlock framework, this study identifies 103 meningioma‐related microbe‐related genes (MRGs), 40 pituitary tumor‐related MRGs, and 63 glioma‐related MRGs expressed in brain tissues. Almost all glioma‐related MRGs are associated with tumor grade and prognosis. Lastly, the prognostic model based on machine learning and microbiota established in this study, namely microbe‐related signature (MRS), could robustly predict the prognosis of glioma and provide insights for immunotherapy benefits. This study presents evidence of the causal effects of gut microbes on brain tumors, which contributes to our understanding of the microbiota‐gut‐brain axis. The relationship between glioma‐related MRGs and glioma prognosis, along with the prognostic prediction capacity of MRS and its association with immunotherapy, provides support for the use of gut microbiota as biomarkers to evaluate the prognosis and treatment response of glioma.

## Graphical abstract

The causal relationship between gut microbiota and brain tumors and potential prognostic value of microbiota are still unclear. This study confirmed the causal effects of specific gut microbiota on three common brain tumors and identified microbe‐related genes that are expressed in brain tissue and correlated with the abundance of gut microbiota. Further, the prognostic model based on machine learning and microbiota established in this study, namely microbe‐related signature, could robustly predict prognosis of glioma and provide insights for immunotherapy benefits.

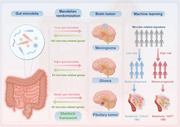

## 1 Introduction

The gut microbiota, comprising trillions of microorganisms inhabiting the gastrointestinal tract [1], has been increasingly recognized as a pivotal environmental factor exerting significant influence on human physiology and pathology [2]. It has been referred to as the “forgotten organ” due to its irrefutable role [3]. In recent years, the discovery of bidirectional communication between the central nervous system and the gastrointestinal tract has led to the prominence of the concept known as the “gut‐brain axis” [4]. The gut microbiota exerts an impact not only on the body's stress level and stress‐related behavior through the gut‐brain axis, but also on brain function [5]. Consequently, there has been a surge of interest among researchers in the field of “microbiota‐gut‐brain axis” [6].

Brain tumors are the leading cause of cancer‐related mortality in individuals of all age groups, including both adults and children [7]. Recent statistics reveal that the worldwide age‐standardized incidence rate and mortality rate for both malignant and non‐malignant brain tumors are approximately 23.41 cases and 4.42 cases per 100,000 population, respectively [8]. The three most common types of brain tumors are meningiomas (39.0%) and pituitary tumors (17.1%) among benign tumors, and glioblastoma (GBM) (14.3%) among malignant tumors, with GBM accounting for half of all malignant brain tumors [8]. Despite prior investigations that have examined and clarified the genetic changes implicated in the advancement of these devastating diseases, existing chemotherapy and targeted treatments still demonstrate no efficacy or offer only marginal enhancements in terms of survival [9]. The unknown cause of brain tumors also makes early diagnosis and treatment difficult. Further exploration into the pathogenesis and prognostic factors of brain tumors represents a pressing concern within this field. The diagnostic and therapeutic significance of gut microbiota in different types of tumors, such as colorectal cancer, has been well‐established [10, 11]. Numerous studies have demonstrated a potential correlation between gut microbiota and brain tumors [12, 13]. For instance, Jiang et al. conducted a study that revealed substantial disparities in the composition and ecological diversity of the gut microbiota between individuals with brain tumors and healthy participants [14]. The composition of the gut microbiota also exhibited significant variations between patients with benign and malignant brain tumors. A case‐control study confirmed the association between oral microbiota and the grade of glioma [15], and that the composition of the gut microbiota is significantly affected by the oral microbiota [16]. However, most current studies regarding gut microbiota and brain tumors are primarily observational studies. As a result, the conclusions drawn from these studies can only establish an “association” rather than “causality” between the two variables, and they are unable to eliminate the potential influence of confounding factors such as diet, medication, and lifestyle choices. In addition, the direction of causation must also be taken into consideration.

Randomized controlled trials help establish cause‐and‐effect relationships. Regrettably, the current limitations in technology and research methods make it challenging to screen specific strains for randomized controlled trials addressing the diagnosis and prognosis aspects. As a result, there is a lack of reported randomized controlled trials investigating the relationship between intestinal microbiota and brain tumors. Mendelian randomization (MR) analysis based on summary data from genome‐wide association studies (GWAS) provides an alternative method to determine causal relationships between exposures and outcomes [17, 18]. MR analysis utilizes genetic variation, usually single‐nucleotide polymorphisms (SNPs), as instrumental variables (IVs), and is based on the random distribution of parental alleles to offspring according to Mendel's law of inheritance. By relying on this randomization, MR analysis can effectively minimize the influence of confounding factors and address issues of reverse causation in studies [19]. Numerous studies have examined the causal link between gut microbiota and human diseases through MR [20, 21]. However, there is a lack of MR studies investigating the potential causal relationship between gut microbiota and brain tumors. Notably, accurate prognostic prediction for patients with brain tumor is essential in advancing personalized treatment, balancing treatment toxicity and survival benefits, and developing rational treatment strategies [22, 23]. Given the importance of the gut‐brain axis and gut microbiota, it may be promising to develop a robust prediction model based on gut microbiota or gut microbes‐related genes.

In the present study, we employed a variety of MR analysis methods to comprehensively analyze the causal relationship between the gut microbiota and the three most common brain tumors. Additionally, we utilized Sherlock, a Bayesian statistical method, to identify genes associated with specific gut microbes in the brain. Moreover, we examined the correlation between microbe‐related genes (MRGs) and clinical characteristics of brain tumors, and integrated numerous machine learning algorithms to construct a robust prognostic model for glioma.

## 2 Methods

**FIGURE 1:**
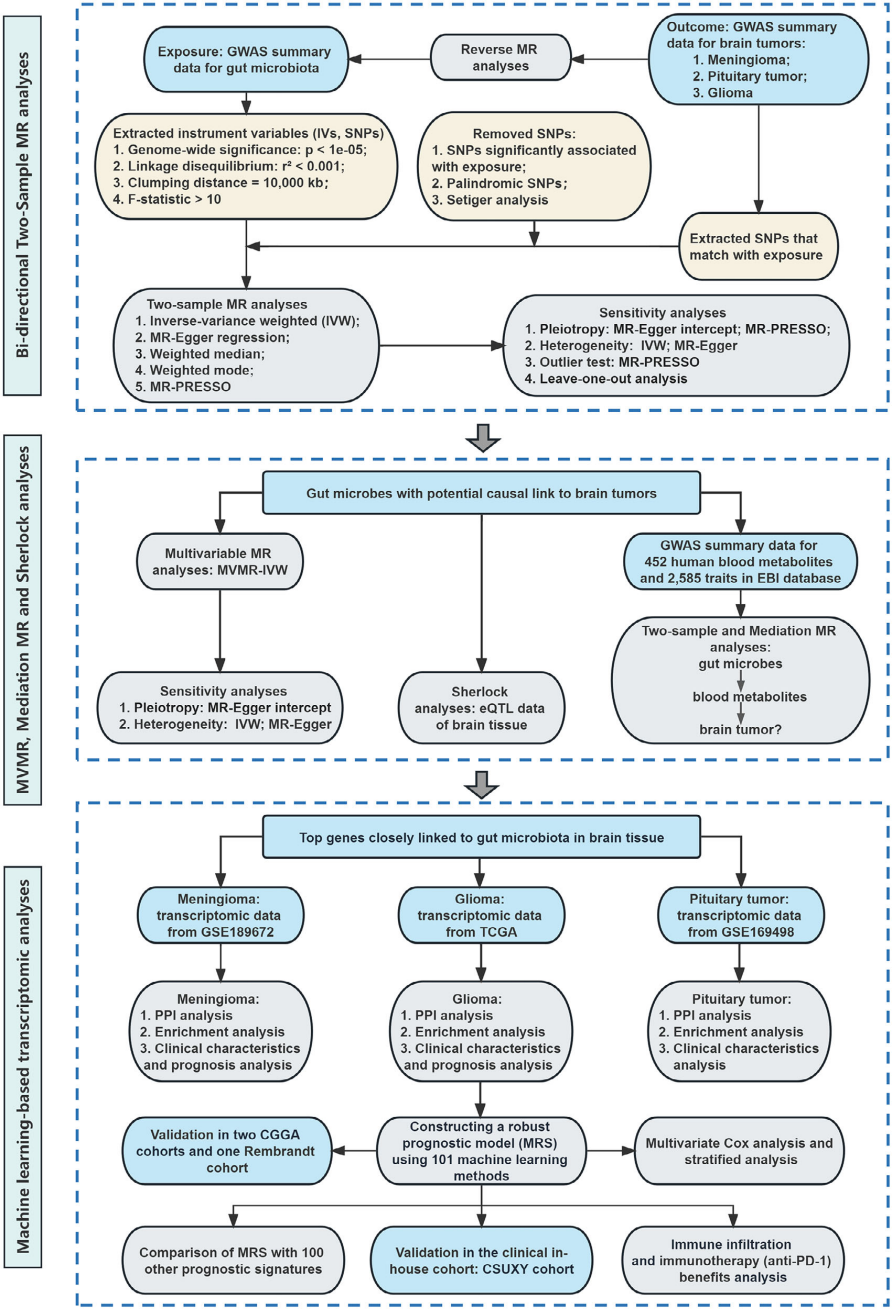
Study design and workflow. The blue blocks represent data sources or analyses that contain data sources. The yellow blocks represent the selection of IVs, and the gray blocks represent the conducted analysis.

The study design of this work was illustrated in Figure 1.

### 2.1 Data Source

For the MR analysis, the data sources and characteristics of GWAS cohorts in this study were summarized in Table S1. The summary data of GWAS for gut microbiota were obtained from the MiBioGen consortium [24]. This large dataset consisted of 18,340 participants from 24 cohorts, incorporating 211 microbial categories. We excluded 15 unknown microbes and finally included 9 phyla, 16 classes, 20 orders, 32 families, and 119 genera, totaling 196 microbial categories. Table S2 provided an overview of each gut microbe included in this study. Summary data for GWAS of human serum metabolites were obtained from the study conducted by Shin et al. The dataset consisted of 7824 European adult participants and covered 452 human serum metabolites [25]. GWAS summary data for both gut microbiota and human serum metabolites are accessible through the IEU Open GWAS (https://gwas.mrcieu.ac.uk/). In addition, we accessed GWAS summary data for 2585 traits in the European Bioinformatics Institute (EBI) database through IEU Open GWAS. The GWAS summary data for human meningiomas, pituitary tumors, and gliomas were obtained from the FinnGen database (https://www.finngen.fi/en) [26].

For the RNA sequencing (RNA‐Seq) data, the GSE189672 cohort [27] containing 110 primary meningioma RNA‐Seq data with complete follow‐up information was collected from the Gene Expression Omnibus database (https://www.ncbi.nlm.nih.gov/geo/). The RNA‐Seq data were transformed into transcripts per kilobase million and further log2(*x* + 1) transformed. Considering that publicly available RNA‐Seq datasets for pituitary tumors lack follow‐up information, we decided to investigate the GSE169498 cohort [28], which includes tumor invasion data and consists of 73 pituitary tumor patients. For glioma, we included a total of 1688 samples from four public cohorts. These cohorts consisted of The Cancer Genome Atlas (TCGA) (*n* = 588) RNA‐Seq cohort, downloaded from UCSC Xena (https://xena.ucsc.edu/), the CGGA1 (*n* = 553) and CGGA2 (*n* = 306) RNA‐Seq cohorts, downloaded from the Chinese Glioma Genome Atlas (CGGA) database (http://www.cgga.org.cn/), and the Rembrandt (*n* = 241) microarray cohort, downloaded from GlioVis (http://gliovis.bioinfo.cnio.es/) [29]. Only samples with complete prognostic information were included, and all data were normalized on the corresponding platform. In addition, we collected 151 eligible glioma samples from the Department of Neurosurgery, Xiangya Hospital, Central South University, Hunan, China for RNA‐Seq to constitute a real‐world clinical in‐house cohort (CSUXY cohort). All patients in the cohort had complete follow‐up data and provided written informed consent. We also obtained a cohort of gliomas treated with PD‐1 inhibitors from the study by Zhao et al [30]. This cohort consisted of 16 GBM samples before anti‐PD‐1 treatment and complete prognostic information after treatment.

### 2.2 RNA Sequencing of Glioma Samples

As described previously [31], total RNA was extracted from the tissue using TRIzol Reagent following the manufacturer's instructions. The quality of the RNA samples was assessed using the 5300 Bioanalyzer (Agilent) and quantified using the ND‐2000 (NanoDrop Technologies). Library preparation and sequencing were performed at Shanghai Majorbio Bio‐pharm Biotechnology Co., Ltd. (Shanghai, China) according to the manufacturer's instructions (Illumina, San Diego, CA). The RNA‐seq transcriptome library was prepared using 1 µg of total RNA and the Illumina Stranded mRNA Prep, Ligation kit. Initially, messenger RNA was isolated using oligo(dT) beads via the polyA selection method and then fragmented using a fragmentation buffer. Next, double‐stranded cDNA was synthesized using a SuperScript double‐stranded cDNA synthesis kit (Invitrogen, CA) with random hexamer primers. The synthesized cDNA underwent end‐repair following Illumina's library construction protocol. After PCR amplification and quantification, the paired‐end RNA‐seq sequencing library was sequenced using the NovaSeq 6000 sequencer (Illumina, San Diego, CA). Then, raw reads were trimmed and quality‐controlled using fastp. The clean reads were aligned to the reference genome using HISAT2. StringTie was used for read assembly, and RSEM (v1.3.3) was used to quantify gene expression levels. Finally, the RNA‐Seq data of 151 glioma samples were converted to TPM and log2 (*x* + 1) transformed to constitute the CSUXY cohort.

### 2.3 Instrumental Variable Selection

SNPs were selected as IVs using a genome‐wide significance threshold of *p* < 1 × 10^−5^ when considering the gut microbiota as the exposure. Based on previously published studies [25, 32], this threshold is the optimal *p* value threshold for selecting genetic predictors associated with microbiome signatures. In order to remove linkage disequilibrium (LD) between IVs, *R* ^2 ^< 0.001 and clumping distance = 10,000 kb were selected as the thresholds for evaluating SNPs during the clumping process. In addition, we excluded SNPs with the *F* statistic <10 to avoid weak instrument bias [33]. Furthermore, we harmonized the SNPs between the exposure and outcome, ensuring that they refer to the same alleles, and removed SNPs strongly associated with the outcome (*p* < 5 × 10^−5^) and palindromic SNPs. To rule out reverse causal association, we performed the Steiger test and excluded SNPs that did not pass the test [34]. For consistency, the same threshold was used to obtain IVs in the reverse two‐sample MR analysis with brain tumors as exposure and gut microbiota as outcome. In the two‐sample MR analysis with gut microbiota as exposure and human serum metabolites as outcome, if the number of matched SNPs with the threshold of *p* < 1 × 10^−5^ is less than three, a threshold of *p* < 5 × 10^−5^ is chosen for SNP selection to ensure the reliability of the analysis. MR‐PRESSO analysis requires more than three SNPs [35]. Default thresholds and parameters were maintained for all other MR analyses.

### 2.4 MR Analysis

The two‐sample MR analysis was used to investigate the causal relationship between exposure and outcome. A total of five MR analysis methods were applied, including inverse variance‐weighted (IVW) [36], MR‐Egger regression [37], weighted median [38], weighted mode [39], and MR‐PRESSO [35]. The IVW method is considered more reliable in certain cases [38]. Therefore, the results obtained using the IVW method will be the primary reference, while the other four methods will be used to complement the IVW analysis.

For sensitivity and heterogeneity analysis, MR‐Egger intercept and MR‐PRESSO were used to determine horizontal pleiotropy. The *P*_intercept_ < 0.05 or the Global Test *p* < 0.05 in MR‐PRESSO indicated the presence of horizontal pleiotropy. In addition, MR‐PRESSO was also used to perform outlier testing. The Cochran's *Q* test of IVW and MR Egger methods was used to assess heterogeneity, with *p* > 0.05 indicating low heterogeneity, and leave‐one‐out analysis was performed to detect SNP outliers.

Due to the interactions among gut microbiota, to assess the independence of causal relationships between different tumor‐related microbes and brain tumors, we employed the IVW method for multivariable MR (MVMR) analysis [39]. We used MR‐Egger intercept to assess pleiotropy of MVMR and IVW and MR Egger to assess heterogeneity. Previous studies have demonstrated that the association between gut microbes and human disease could be mediated by serum metabolites [6, 40, 41, 42]. Therefore, we hypothesized that the influence of gut microbes on brain tumors is potentially mediated by serum metabolites. To explore this hypothesis, two‐sample MR analysis was first used to identify serum metabolites that are causally linked to specific tumor‐related microbes. Subsequently, we performed the two‐step MR (TSMR) analysis to examine the mediating role of serum metabolites. In addition to obtaining the total effect (*c*) of microbes on brain tumors through two‐sample MR, TSMR also needs to obtain the causal effect of gut microbes on the mediator (*a*) and the causal effect of the mediator on brain tumors (*b*). The mediation effect was determined using the coefficient product method (*a *× *b*), and the proportion of the mediation effect was calculated as “indirect effect/total effect” ([a × b]/c). The significance of the mediation effect was evaluated using interactive mediation test. This method was also used to explore potential mediators among the 2585 traits of the EBI database.

### 2.5 Identification of Specific Tumor Microbe‐related Genes in the Brain

The Sherlock (http://sherlock.ucsf.edu/index.html) [43] was used to identify genes expressed in the brain that are associated with specific tumor‐related microbes. Sherlock uses Bayesian statistical methods to match gene signatures in expression quantitative trait loci (eQTL) with GWAS data. Compared to other methods, Sherlock integrates information from all eQTL SNPs, including cis‐ and trans‐, and is capable of collecting easily overlooked clues from GWAS. It can distinguish causality from coincidence and is applicable to any molecular trait. According to Sherlock's instructions, we uploaded the GWAS data of specific gut microbe and selected the eQTL data of the cerebral cortex in the GTEx (V7) database for analysis. The top associated genes evaluated by Sherlock were then selected from the analysis results as MRGs for the specific microbe.

### 2.6 Protein–Protein Interaction Analysis and Enrichment Analysis

The protein–protein interaction (PPI) analysis was performed using STRING (https://string‐db.org/). MRGs from three types of brain tumors were separately input into STRING, and PPI networks were constructed using default parameters. In addition, the R package “clusterProfiler” was used to perform Kyoto Encyclopedia of Genes and Genomes (KEGG) pathway analysis and Gene Ontology (GO) biological processes enrichment analysis on MRGs in three types of brain tumors.

### 2.7 Constructing a Prognostic Model for Glioma Based on Comprehensive Machine Learning Approaches

To develop a robust and accurate prognostic model, namely microbe‐related signature (MRS), for glioma, we adopted 101 algorithm combinations based on ten machine learning algorithms. The machine learning algorithms used include CoxBoost, partial least squares regression for Cox (plsRcox), supervised principal components, Ridge, elastic network, stepwise Cox, random survival forest, Lasso, survival support vector machine and generalized boosted regression modelling. First, common MRGs from four glioma public cohorts (TCGA, CGGA1, CGGA2, and Rembrandt) were extracted. Then, the expression matrix of common MRGs from the TCGA cohort was extracted, and 101 algorithm combinations were used to fit the prediction model based on the leave‐one‐out cross‐validation framework for MRGs in the TCGA cohort [44]. Then we evaluated all models across four cohorts and calculated the average C‐index for each model. The model combination with the highest C‐index was considered the best model, Similar to previous studies [45, 46], multivariate Cox regression was performed on the MRGs in the best model and risk scores were calculated by applying the Cox regression coefficients weighted gene expression of MRGs.

### 2.8 Immune Infiltration Analysis

According to previous studies [47, 48], the infiltration abundance of 28 immune cells in each glioma sample was quantified using the single‐sample gene set enrichment analysis method.

### 2.9 Statistical Analysis

All statistical calculations were performed using R software (version 4.3.1). All MR analyses were performed using the TwoSampleMR (version 0.5.7), MendelianRandomization (version 0.8.0), and MRPRESSO package (1.0). In MR analysis, *p* < 0.05 is considered to have a suggestive causal association. Since this is an exploratory study, adjusted *p*‐values were not used. Differences between two groups were compared using unpaired Student's *t*‐test or Wilcoxon rank sum test. For comparisons between more than two groups, differences were compared using one‐way ANOVA or Kruskal–Wallis test. The “survminer” package was used to determine the optimal cutoff values for survival analysis, while “survival” package was used to generate Kaplan–Meier (KM) curves and perform log‐rank tests to determine significance. The “pROC” package was used to plot receiver operating characteristic (ROC) curves and calculate the area under the curve (AUC). Univariate Cox regression was employed to assess the prognostic significance of individual genes. Due to the substantial collinearity between the WHO grade and histological characteristics of gliomas, only histological characteristics were used as covariates in the multivariate Cox regression analysis. *P* values were two‐sided and *p* < 0.05 was regarded as statistically significant.

## 3 Results

### 3.1 Causal Effects of Gut Microbiota on Three Brain Tumors

**FIGURE 2:**
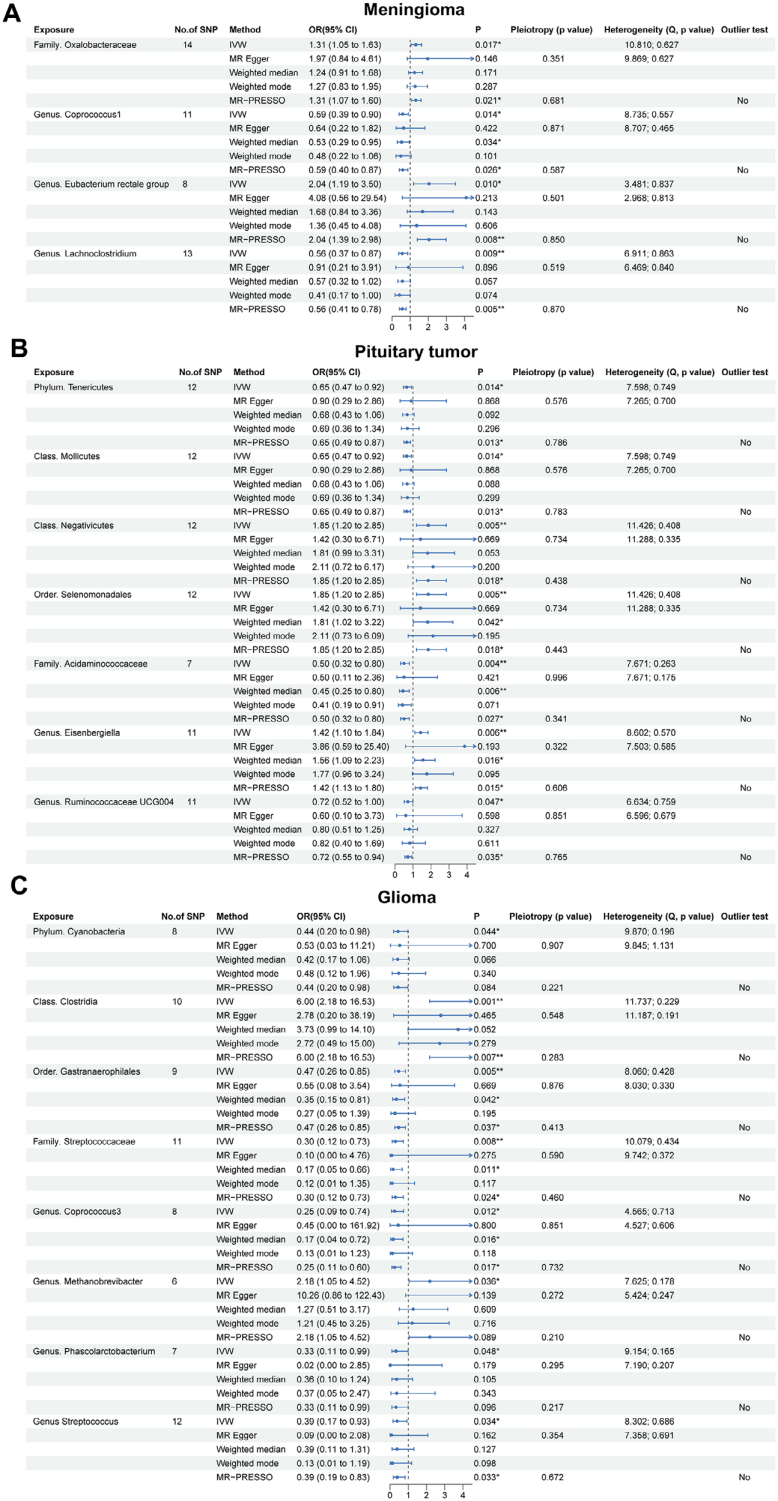
Two sample MR results of causal effects of gut microbes on meningioma (A), pituitary tumor (B), and glioma (C). Data are expressed as an odds ratio (OR) with corresponding 95% confidence interval (CI). The forest plots also include the results of sensitivity analyses. *p < 0.05, **p < 0.01.

In order to explore the causal effects of gut microbiota on common brain tumors, the two‐sample MR analysis was conducted and all results were summarized in Tables S3–S5. As shown in Figure 2A, for meningioma, the IVW analysis indicated that a higher abundance of family *Oxalobacteraceae* (OR = 1.31, 95%CI = 1.05∼1.63, *p* = 0.017) and genus *Eubacterium rectale group* (OR = 2.04, 95%CI = 1.19∼3.50, *p* = 0.010) has potential causal effects with meningioma. The lower abundance of genus *Coprococcus1* (OR = 0.59, 95%CI = 0.39∼0.90, *p* = 0.014) and genus *Lachnoclostridium* (OR = 0.56, 95%CI = 0.37∼0.87, *p* = 0.009) exhibited potential causal effects on meningioma. For pituitary tumor, IVW analysis revealed that class *Negativicutes* (OR = 1.85, 95%CI = 1.20∼2.85, *p* = 0.005), order *Selenomonadales* (OR = 1.85, 95%CI = 1.20∼2.85, *p* = 0.005), and genus *Eisenbergiella* (OR = 1.42, 95%CI = 1.10∼1.84, *p* = 0.006) have positive causal effects on pituitary tumors. On the other hand, phylum *Tenericutes* (OR = 0.65, 95%CI = 0.47∼0.92, *p* = 0.014), class *Mollicutes* (OR = 0.65, 95%CI = 0.47∼0.92, *p* = 0.014), family *Acidaminococcaceae* (OR = 0.50, 95%CI = 0.32∼0.80, *p* = 0.004), and genus *Ruminococcaceae UCG004* (OR = 0.72, 95%CI = 0.52 ∼1.00, *p* = 0.047) have negative effects on pituitary tumors (Figure 2B). For glioma, IVW analysis indicated that the increased abundance of class *Clostridia* (OR = 6.00, 95%CI = 2.18∼16.53, *p* = 0.001) and genus *Methanobrevibacter* (OR = 2.18, 95%CI = 1.05∼4.52, *p* = 0.036) may be causally linked to gliomas. Conversely, the decreased abundance of phylum *Cyanobacteria* (OR = 0.44, 95%CI = 0.20∼0.98, *p* = 0.044), order *Gastranaerophilales* (OR = 0.47, 95%CI = 0.26∼0.85, *p* = 0.005), family *Streptococcaceae* (OR = 0.30, 95%CI = 0.12∼0.73, *p* = 0.008), genus *Coprococcus3* (OR = 0.25, 95%CI = 0.09∼0.74, *p* = 0.012), genus *Phascolarctobacterium* (OR = 0.33, 95%CI = 0.11∼0.99, *p* = 0.048), and genus *Streptococcus* (OR = 0.39, 95%CI = 0.17∼0.93, *p* = 0.034) may also be causally linked to gliomas (Figure 2C). The results of the other four methods support the findings of the IVW method. In addition, various sensitivity analysis methods did not find potential horizontal pleiotropy or heterogeneity (*p* > 0.05) (Figure 2A–C). The leave‐one‐out analysis also did not detect any outlier SNPs (Figures S1–S3).

### 3.2 MVMR Analysis and Mediation MR Analysis of Gut Microbiota

Considering that the abundance of gut microbes may be affected by each other, we conducted MVMR‐IVW analysis on gut microbiota that may potentially have a causal association with three types of brain tumors to explore the significance of distinct microbe in the causal effect. In meningiomas, the causal effect of genus *Coprococcus1* (*p* = 0.192) on meningiomas is no longer significant when adjusting for four microbes using MVMR‐IVW (Table 1), suggesting that genus *Coprococcus1* may be affected by the abundance of the other three microbes. For pituitary tumors, when adjusting the seven microbes together using MVMR‐IVW, the causal effects of all microbes are still significant (Table 1), suggesting that these microbes independently contribute to the causation of pituitary tumors. In glioma, when adjusting for eight microbes together, only class *Clostridia* (*p* = 0.008) remains significant (Table 1), suggesting that class *Clostridia* may play a dominant role in the causal effect of these microbes on gliomas. Additionally, the results of MVMR‐Egger indicate that our MVMR‐IVW analyses are unlikely biased by pleiotropy (*P*_intercept_ > 0.05).

**TABLE 1:** Multivariable MR results of the causal effect of gut microbiota on brain tumors.

Exposure	No. of SNP	Method	OR	95%CI	*p* value	Egger intercept, *p* value	Heterogeneity (*Q*, *p* value)
**Meningioma**							
Family. *Oxalobacteraceae*	41	IVW	1.28	1.03∼1.59	0.028	0.644	IVW (24.79, 0.937); Egger (24.58, 0.925)
	
Genus. *Coprococcus*1		IVW	0.71	0.42∼1.18	0.192		
Genus. *Eubacterium rectale* group		IVW	1.90	1.14∼3.17	0.014		
Genus. *Lachnoclostridium*		IVW	0.56	0.37∼0.86	0.007		
**Pituitary tumor**							
Phylum. *Tenericutes*	47	IVW	3.11	1.91∼5.07	<0.001	0.935	IVW (25.13, 0.968); Egger (25.09, 0.959)
Class. *Mollicutes*		IVW	0.59	0.41∼0.87	0.008		
Class. *Negativicutes*		IVW	3.11	1.91∼5.07	<0.001		
Order. *Selenomonadales*		IVW	0.59	0.41∼0.87	0.008		
Family. *Acidaminococcaceae*		IVW	0.37	0.23∼0.59	<0.001		
Genus. *Eisenbergiella*		IVW	1.55	1.21∼1.99	<0.001		
Genus. *Ruminococcaceae* UCG004		IVW	0.72	0.52∼0.98	0.037		
**Glioma**							
Phylum. *Cyanobacteria*	48	IVW	3.49	0.21∼57.8	0.383	0.908	IVW (66.23, 0.005); Egger (66.21, 0.004)
Class. *Clostridia*		IVW	6.26	1.61∼24.3	0.008		
Order. *Gastranaerophilales*		IVW	0.23	0.02∼2.48	0.224		
Family. *Streptococcaceae*		IVW	0.01	0∼399.17	0.389		
Genus. *Coprococcus*3		IVW	0.35	0.07∼1.79	0.205		
Genus. *Methanobrevibacter*		IVW	1.66	0.82∼3.37	0.159		
Genus. *Phascolarctobacterium*		IVW	0.68	0.25∼1.85	0.445		
Genus. *Streptococcus*		IVW	39.20	0∼129.00	0.490		

Given the possible contribution of serum metabolites to the causal relationship between gut microbiota and brain tumors [6, 40, 41, 42], we integrated two‐sample MR and TSMR analyses to investigate the potential mediation effects of serum metabolites. First, we performed two‐sample MR with serum metabolites as the outcome, and identified a total of 20 serum metabolites that are causally related to four meningioma‐related microbes, including pyruvate (Table S6). In addition, 25 serum metabolites with potential causal links to pituitary tumor‐related microbes and 49 serum metabolites with potential causal links to glioma‐related microbes were identified (Tables S7 and S8). However, the TSMR analysis conducted using these serum metabolites as mediators did not yield any positive results, indicating that brain tumor‐related microbes may not affect the occurrence of brain tumors through these serum metabolites (Tables S6–S8). In order to expand the search range of mediators, we conducted the TSMR analyses on 2585 traits in the EBI database. It was found that the ratio of lysophosphatidylcholine (LPC) to lysophosphatidylethanolamine (LPE) is a potential mediator of the causal effect of family *Oxalobacteraceae* on meningiomas (Table S9). Family *Oxalobacteraceae* may promote the occurrence of meningiomas by reducing LPC/LPE, with a mediation effect accounting for 51.2%.

### 3.3 Identification of Microbe‐Related Genes in the Brain

Considering the bidirectional communication of the gut‐brain axis, the abundance of gut microbiota may be influenced by brain tumors [4]. We conducted reverse two‐sample MR analysis based on GWAS data of brain tumor, but there is no evidence that the three brain tumors could have a causal effect on the abundance of gut microbiota (Tables S10–S12). Gene expression data based on eQTL may provide more information, and we performed Sherlock analysis to identify genes in the brain that influence the abundance of specific gut microbes. For the four microbes associated with meningiomas, we identified a total of 103 MRGs, which we refer to as meningioma‐related MRGs (Table S13). In addition, we identified a total of 40 pituitary tumor‐related MRGs among seven microbes associated with pituitary tumors, and a total of 63 glioma‐related MRGs among eight microbes associated with gliomas (Table S13). PPI analysis revealed that meningioma‐related MRGs formed a closely interconnected interaction network (Figure S4A), while pituitary tumor‐related MRGs and glioma‐related MRGs both showed fewer interactions (Figure S4B,C). Biological annotation through GO and KEGG analysis found that meningioma‐related MRGs are mainly involved in cellular catabolic processes and autophagy (Figure S4D), and pituitary tumor‐related MRGs are mainly related to the movement of cell or subcellular component, protein phosphorylation, and bacterial invasion of epithelial cells (Figure S4E). Glioma‐related MRGs are mainly enriched in symbiotic processes, endomembrane system organization and endosomal transport (Figure S4F).

### 3.4 The Association Between Microbe‐Related Genes and Clinical Characteristics of Brain Tumors

**FIGURE 3:**
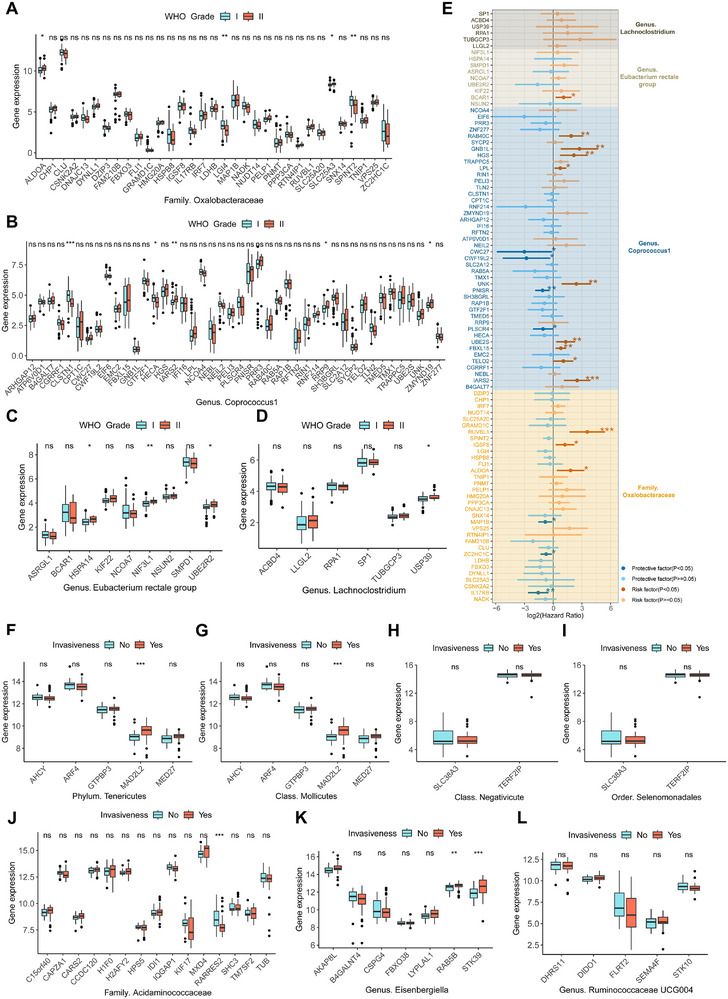
The relationship between MRGs in meningiomas and pituitary tumors and clinical characteristics. (A–D) The expression differences of meningioma‐related MRGs between WHO grade I and II meningiomas. Figures (A–D) respectively represent the expression of MRGs corresponding to the gut microbe that has a causal effect on meningiomas. (E) The forest plot displays the results of univariate Cox regression analyses of meningioma‐related MRGs on the recurrence‐free survival of meningiomas. The different colored squares correspond to MRGs of different gut microbes. (F–L) The expression differences of pituitary tumor‐related MRGs between invasive and non‐invasive pituitary tumors. Figures (F–L), respectively represent the expression of MRGs corresponding to the gut microbe that have a causal effect on pituitary tumors. *p < 0.05, **p < 0.01, ***p < 0.001.

To further explore the potential clinical significance of MRGs in brain tumors, we collected multiple transcriptomic cohorts of brain tumors. In the meningioma cohort (GSE189672), it was found that the MRGs corresponding to each microbe with a causal effect on meningiomas all have specific genes associated with the WHO grade of meningiomas (Figure 3A–D). For example, ALDOA, associated with the family *Oxalobacteraceae*, is expressed at higher levels in grade II meningiomas (Figure 3A). However, only in MRGs of the family *Oxalobacteraceae* and the genus *Coprococcus1*, there are genes associated with the recurrence of meningiomas after surgical resection (Figure S5). In terms of prognostic value, multiple meningioma‐related MRGs were found to be closely associated with the recurrence‐free survival of meningiomas (Figure 3E), indicating the feasibility of these MRGs as prognostic biomarkers. In the pituitary tumor cohort (GSE169498), we identified a total of five MRGs (MED27, RARRES2, AKAP8L, RAB5B, and STK39) that are associated with the invasiveness of pituitary tumors (Figure 3F–L).

**FIGURE 4:**
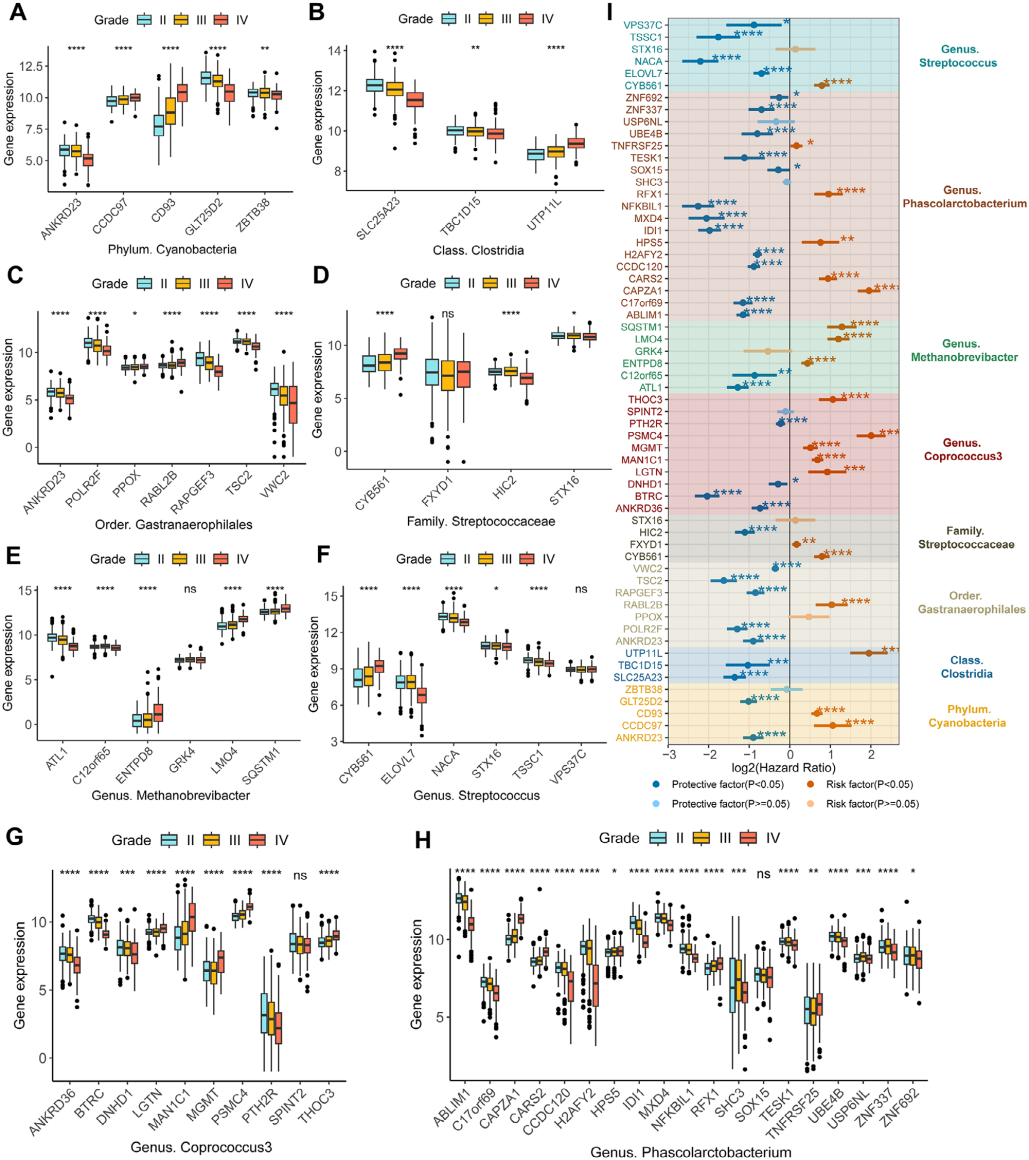
The relationship between MRGs in gliomas and clinical characteristics. (A–H) The expression differences of glioma‐related MRGs between different WHO grades. Figures (A–H) respectively represent the expression of MRGs corresponding to the gut microbe that have a causal effect on gliomas. (I) The forest plot displays the results of univariate Cox regression analyses of glioma‐related MRGs on the OS of gliomas. The different colored squares correspond to MRGs of different gut microbes. *p < 0.05, **p < 0.01, ***p < 0.001, ****p < 0.0001.

For glioma, the most common malignant brain tumor, we first explored the association between MRGs and the clinical characteristics of glioma using the TCGA cohort. Surprisingly, apart from SOX15, all MRGs are significantly correlated with the WHO grade of glioma (Figure 4A–H). Additionally, almost all MRGs are closely associated with the overall survival (OS) of glioma (Figure 4I), indicating the potential significance of MRGs in the advancement and prognosis of glioma. Since the expression of MRGs serves as an indirect indicator of the abundance of gut microbes, it also implies that the abundance of gut microbiota might impact the prognosis of glioma.

### 3.5 The Construction of a Robust Microbe‐Related Signature Based on Machine Learning

**FIGURE 5:**
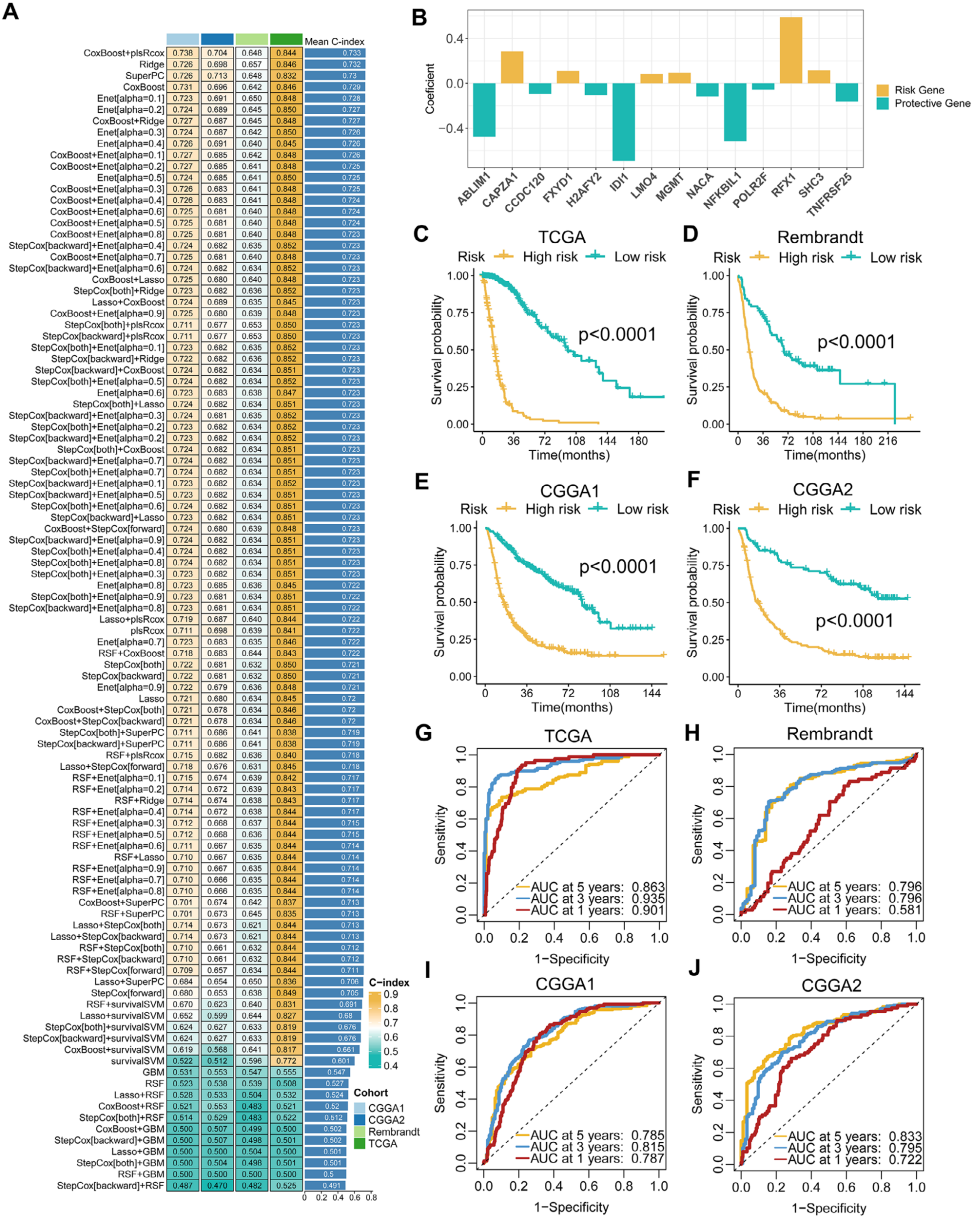
The establishment and validation of the MRS model via the machine learning‐based integrative procedure. (A) The 101 algorithm combination models of the integrated framework of machine learning and the C‐index of each model on all cohorts. (B) Coefficients of multivariable Cox regression analysis of 14 key MRGs finally obtained in CoxBoost+plsRcox model. (C–F) Kaplan–Meier curves depict the OS difference of glioma between MRS‐high and MRS‐low groups in TCGA (C), Rembrandt (D), CGGA1 (E), and CGGA2 (F) cohorts. Yellow represents the MRS‐high group, and green represents the pathway MRS‐low group. (G–J) ROC curves showing the OS prediction efficiency of MRS in TCGA (G), Rembrandt (H), CGGA1 (I), and CGGA2 (J) glioma cohorts.

Currently, there is no established follow‐up cohort focusing on gut microbiota in glioma. The strong correlation between MRGs and glioma grade and prognosis has sparked our enthusiasm, prompting us to explore the feasibility of using MRGs to construct a reliable prognostic model (namely microbe‐related signature, MRS) as an alternative to gut microbe models. To develop the MRS, we integrated the expression profiles of 63 glioma‐related MRGs from four cohorts, including 1688 samples. Then, we fitted 101 algorithm combinations based on 10 machine learning algorithms using the TCGA cohort as the training dataset, and further calculated the (C‐index of each algorithm combination across all cohorts (Figure 5A). Strikingly, the algorithm combination of CoxBoost+plsRcox consistently exhibited a superior C‐index across all cohorts and attained the highest average C‐index (0.733). The optimal model CoxBoost+plsRcox identified 14 key prognostic‐related MRGs. Subsequently, the regression coefficients of each MRG were calculated using a multivariate Cox regression model (Figure 5B), and the expression of the 14 MRGs weighted by coefficients was used to calculate the risk score for each patient. According to the optimal cutoff value, patients from the four public cohorts were divided into high‐risk and low‐risk groups. The KM curves show that patients in the high‐risk group had significantly worse OS (Figure 5C–F, all *p* < 0.0001), both in the training dataset (TCGA) and validation datasets (Rembrandt, CGGA1 and CGGA2). According to differences in molecular pathology and malignant behavior, gliomas can be broadly divided into GBM and non‐GBM [49]. Further analysis demonstrates that high‐risk patients also have worse OS in both GBM and non‐GBM (Figure S6, all *p* < 0.05). Multivariate Cox analysis shows that MRS remains statistically significant after adjusting for age, gender, histological characteristics and IDH status (Figure S7, all *p* < 0.001), indicating that MRS is an independent risk factor for glioma. Subsequently, the discriminatory ability of MRS is evaluated using ROC curves. The AUCs of 1‐year, 3‐year, and 5‐year OS in TCGA cohort are 0.901, 0.935, and 0.863, respectively; in Rembrandt cohort, they are 0.863, 0.935, and 0.901; in CCGA1 cohort, they were 0.787, 0.815, and 0.785; in CGGA2 cohort, they are 0.722, 0.795, and 0.835 (Figure 5G–J). Overall, these results indicate that MRS has accurate and robust predictive ability in multiple independent cohorts.

### 3.6 Validation of MRS in the Clinical In‐House Cohort and Comparison of Prognostic Signatures

**FIGURE 6:**
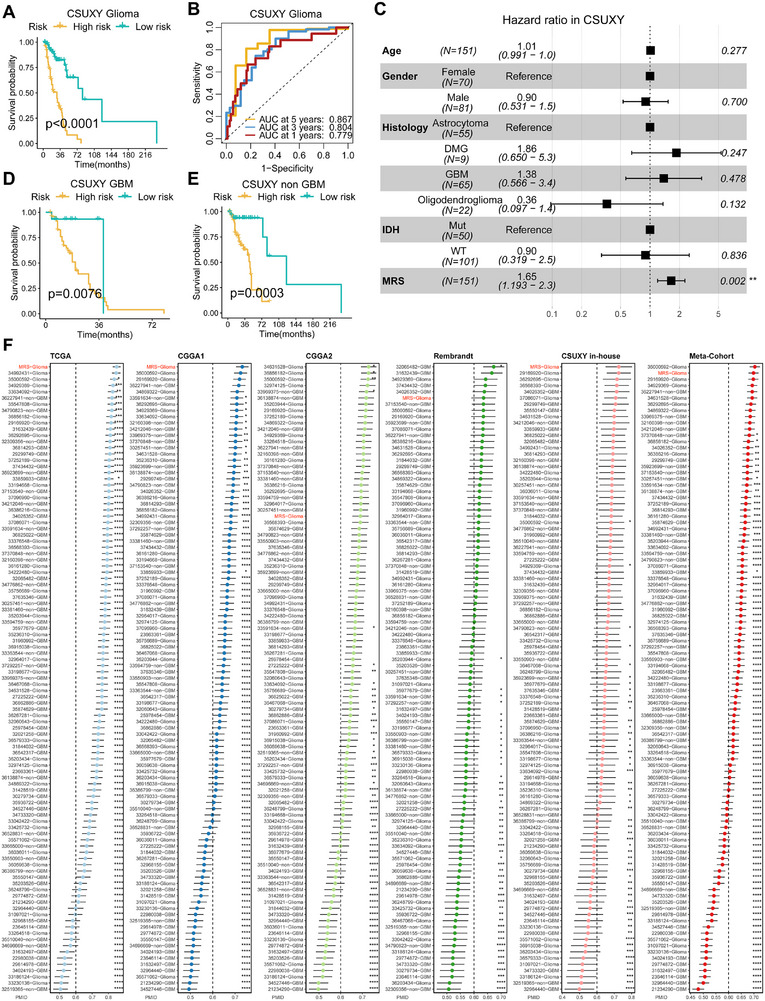
Validation of MRS in the clinical in‐house cohort and comparison of prognostic signatures. (A) Kaplan–Meier curve depicts the OS difference of glioma between MRS‐high and MRS‐low groups in the CSUXY in‐house cohort. (B) ROC curve showing the OS prediction efficiency of MRS in the CSUXY in‐house glioma cohort. (C) Multivariable Cox regression analysis of OS in the CSUXY in‐house cohort. Statistic test: two‐sided Wald test. Data are presented as hazard ratio (HR)  ±  95% confidence interval (CI). (D) Kaplan–Meier curve depicts the OS difference of GBM between MRS‐high and MRS‐low groups in the CSUXY in‐house cohort. (E) Kaplan–Meier curve depicts the OS difference of non‐GBM between MRS‐high and MRS‐low groups in the CSUXY in‐house cohort. (F) C‐index analysis MRS and 100 published signatures in TCGA, CGGA1, and CGGA2, Rembrandt, CSUXY in‐house, and meta‐cohort. Statistic tests: Two‐sided *z*‐score test. Data are presented as mean ± 95% confidence interval (CI). *p < 0.05; **p < 0.01; ***p < 0.001; ****p < 0.0001.

Given the reliable performance of MRS in public cohorts, we further conducted RNA‐Seq analysis on 151 glioma samples from the clinical in‐house cohort (CSUXY) to facilitate the clinical translation and application of the MRS model. As shown in Figure 6A, the KM curve demonstrates that the high‐risk group still has a significantly worse OS than the low‐risk group in the in‐house cohort. The ROC curve shows that the AUCs of 1‐year, 3‐year, and 5‐year OS in the in‐house cohort are 0.779, 0.804, and 0.867, respectively (Figure 6B), which suggests the excellent predictive performance of MRS. Furthermore, we conducted multivariate Cox regression analysis with age, gender, histological characteristics, and IDH status as covariates. The results show that the MRS model remains statistically significant for OS (Figure 6C, *p* = 0.002), indicating that MRS is also an independent risk factor in the in‐house cohort. Stratified analysis confirms that MRS has prognostic value in both the GBM and non‐GBM cohorts (Figure 6D,E, all *p* < 0.01), which is consistent with previous findings.

With the popularity of RNA‐Seq technology, numerous gene expression‐based tumor prognostic signatures have been developed. To compare the efficacy of the MRS model with other gene signatures, we searched a large number of recently published signatures. Given that MRS is a prognostic model based on mRNA expression, we eventually enrolled 100 published mRNA signatures in gliomas (Table S14). These signatures involve various important tumor‐related biological processes such as immune response, glycolysis, and autophagy. After comparing the C‐index of the MRS model with other signatures, it was found that the MRS model not only has the best performance in the training dataset (TCGA) but also displays the best performance in the CGGA1 and CSUXY cohorts (Figure 6F). Moreover, MRS also shows nearly the best performance in the Rembrandt cohort and the meta‐cohort that integrated all validation datasets (Figure 6F). Despite its relatively poorer performance in the CGGA2 cohort, MRS still outperforms most other signatures (Figure 6F). It is worth noting that some signatures performed well in the CGGA2 cohort but poorly in other cohorts, which could be attributed to their overfitting in the CGGA2 cohort.

### 3.7 The Association Between MRS and Immune Infiltration as Well as Immunotherapy Response in Glioma

**FIGURE 7:**
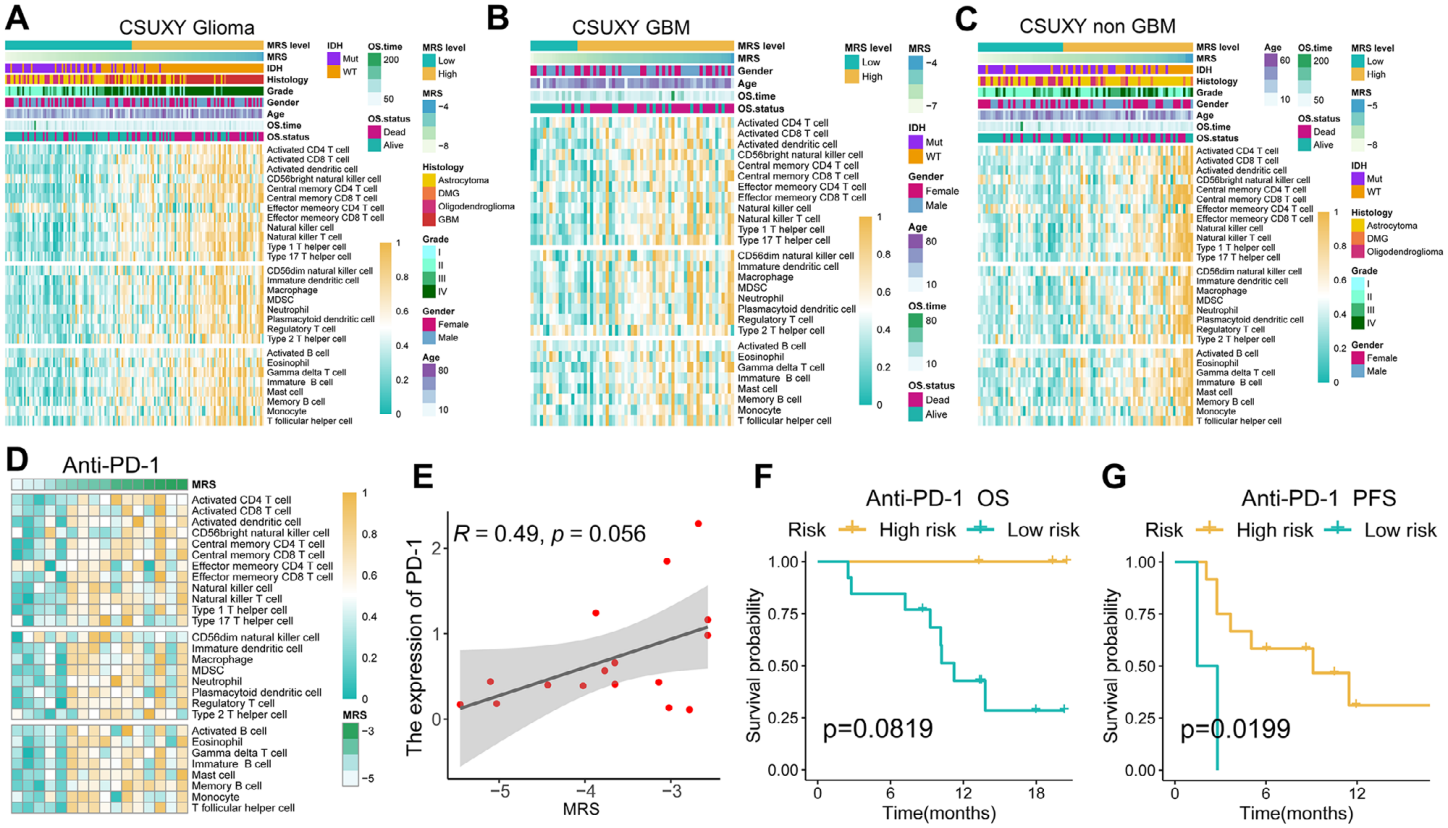
The association between MRS and immune infiltration as well as immunotherapy response in glioma. (A–C) Heatmaps of the relationship between MRS and 28 immune cells in glioma (A), GBM (B), and non‐GBM (C) of the CSUXY in‐house cohort. Age, gender, OS status, OS time, grade, histology, and IDH status are shown as patient annotations. (D) Heatmap of the relationship between MRS and 28 immune cells in the anti‐PD‐1 cohort. (E) Correlation between MRS and the expression of PD‐1 in the anti‐PD‐1 cohort. Statistic test: Pearson's correlation coefficient. (F, G) Kaplan–Meier curves depict the overall survival (F) and progression‐free survival (G) differences of GBM between MRS‐high and MRS‐low groups in the anti‐PD‐1 cohort.

MRS is a model based on gut microbe‐related mRNA and previous studies in the last decade have emphasized the importance of the interaction between gut microbiota and the immune system [50, 51, 52]. Therefore, we hypothesized that MRS may be associated with the immune characteristics and immunotherapy efficacy of glioma. In the CSUXY in‐house cohort, immune cell infiltration analysis indicates positive correlations between MRS and the infiltration levels of immune cells in glioma (Figure 7A), including anti‐tumor immune‐stimulating cells (such as activated CD8 T cells) and pro‐tumor immune‐suppressive cells (such as tumor‐associated macrophages). Consistent results were also observed in the TCGA, CGGA1, CGGA2, and Rembrandt cohorts (Figure S8). After further dividing the CSUXY cohort into GBM and non‐GBM groups, MRS still showed positive correlations with the levels of immune cell infiltration in both the GBM and non‐GBM groups (Figure 7B,C). These results imply that patients with high MRS have a relatively “hot” tumor microenvironment (TME). It is known that pre‐existing anti‐tumor immunity is favorable for the clinical benefits of immune checkpoint inhibitor (ICI) [53], therefore, we speculated that patients with high MRS may benefit more from ICI therapy. We further included a GBM anti‐PD‐1 treatment cohort from a previous study [30], in which MRS still shows positive correlations with the levels of immune cell infiltration in tumors (Figure 7D). Additionally, MRS is also positively correlated with PD‐1 expression in the anti‐PD‐1 cohort (Figure 7E). KM curve analysis demonstrates that patients with high MRS exhibit prolonged OS (Figure 7F, *p* = 0.0819) and progression‐free survival (Figure 7G, *p* = 0.0199) following treatment with anti‐PD‐1 therapy, suggesting a higher benefit from anti‐PD‐1 therapy, which is consistent with our hypothesis.

## 4 Discussion

The emergence of the microbiota‐gut‐brain axis suggests a new possibility for tumor treatment [6, 54]. Recent research has confirmed, from the perspectives of animal models and case‐control studies, the alterations of gut microbiota in patients with brain tumors and their association with clinical pathological characteristics [14, 15, 55, 56]. Our study systematically analyzed the bidirectional causal relationship between gut microbiota and the three most common brain tumors from a genetic perspective. Various MR analysis methods based on GWAS have provided compelling evidence for the non‐negligible role of gut microbiota in the onset and progression of brain tumors. We also employed transcriptome‐wide association study (TWAS), which integrated population‐level transcriptome data with GWAS summary data to identify MRGs. Furthermore, we combined bulk RNA‐Seq data with comprehensive machine learning methods to analyze the clinical relevance of these MRGs. To the best of our knowledge, this is the first work that systematically explores the potential association between gut microbiota and brain tumors by integrating multidimensional data such as GWAS, TWAS, and bulk RNA‐Seq.

In the forward two‐sample MR analysis, we identified four gut microbes with causal effects on meningioma. Family *Oxalobacteraceae* and genus *Eubacterium rectale group* may be risk factors for meningioma, while genus *Coprococcus* and genus *Lachnoclostridium* may be protective factors. Currently, we have only found one study investigating microbial changes in meningioma patients, which found a decrease of genus *Lachnospira* in them [14]. Genus *Lachnospira*, genus *Coprococcus*, and genus *Lachnoclostridium* all belong to the family *Lachnospiraceae*, which may suggest an important protective role of the family *Lachnospiraceae* in meningioma. Numerous studies have demonstrated that *Lachnospiraceae* bacteria are important probiotics in the human body [57, 58], known for their production of sodium butyrate. Recent research has further highlighted the significant role of these bacteria in tumor suppression [59, 60, 61], which aligns with our study. Interestingly, it is observed that the protective effect of the genus *Coprococcus* is diminished in the MVMR analysis, whereas the effect of the genus *Lachnoclostridium* is amplified, which highlights the potentially more pivotal role of the genus *Lachnoclostridium*. In addition, family *Oxalobacteraceae*, as a meningioma risk factor in our study, has also been proven to be enriched in high‐grade colorectal cancer [62]. In terms of pituitary tumors, this study found that phylum *Tenericutes*, class *Negativicutes*, and genus *Eisenbergiella* are risk factors, while class *Mollicutes*, order *Selenomonadales*, family *Acidaminococcaceae*, and genus *Ruminococcaceae UCG00*4 are protective factors. Class *Mollicutes* belongs to phylum *Tenericutes*, which may explain their similar efficacy. Similarly, order *Selenomonadales* belongs to class *Negativicutes*, and they also exhibit similar efficacy. Previous studies have demonstrated a higher abundance of phylum *Tenericutes* in patients with colorectal adenocarcinoma compared to healthy individuals [63] and this phylum has been implicated in breast carcinogenesis in both sexes [64]. A report indicates that class *Negativicutes* significantly increases in the oral microbiota of patients with esophageal cancer [65]. Zhang et al. demonstrated the association of genus *Eisenbergiella* with poorly differentiated colorectal cancer with higher malignancy [66]. These findings support the role of these microbes as risk factors for pituitary tumors. In line with this, there is evidence showing a decreased abundance of *Ruminococcaceae* bacteria in endometrial cancer patients [67], providing further support for the concept of *Ruminococcaceae* bacteria as protective factors. Gliomas have received more attention from researchers compared to benign tumors. The relationship between gut microbiota and glioma was first investigated in a study conducted by Kim et al. in 2016 [68]. This study discovered that metabolites derived from gut microbiota can suppress cell migration in glioma cells by reducing the expression of MMP2 and MMP9. Multiple studies have confirmed the variations of *Firmicutes* abundance in both mouse models and patients with glioma [14, 69], as well as their potential correlation with chemotherapy [56, 69, 70], however, conflicting findings exist. Patrizz et al. and Jiang et al. provide evidence for the reduction of *Firmicutes* in glioma [14, 69], whereas Fan et al. suggests an enrichment of *Firmicutes* [70]. The reasons for the heterogeneity in the results may stem from limitations in sample size and variations in the models used. The class *Clostridia*, which belongs to the phylum *Firmicutes*, was found to be a risk factor for glioma in two sample MR analysis of this study. Furthermore, the MVMR analysis underscored the importance of the class *Clostridia*, indirectly confirming the enrichment of the phylum *Firmicutes* in gliomas. This study has also identified specific microbes that exhibit potential causal effects on gliomas, which have not been previously investigated in glioma research. One notable example is the genus *Coprococcus*, which has been found to have a protective effect on both gliomas and meningiomas, making it a significant probiotic candidate. It is worth noting that, given the complexity and dynamics of the gut microbiota, further establishing new cohorts to verify these complex causal relationships can effectively reduce bias and is meaningful. This study identified specific microbes at the genetic level that exhibited causal effects on brain tumors. These microbes warrant further investigation and represent potential candidates for microbial therapy in the prevention and treatment of brain tumors. Employing dietary strategies to target these gut microbes could be an effective approach [71]. In addition, fecal microbiota transplantation (FMT), as a direct intervention method, can regulate the gut microbiota as a whole and shows a promising foreground [72]. Previously published clinical trial results have demonstrated that FMT can improve resistance to immunotherapy in cancer patients through long‐term modulation of gut microbiota [73]. We believe that incorporating gut microbiota assessment as part of routine clinical check‐ups and performing FMT for individuals at microbiota dysbiosis could be a meaningful strategy to potentially lower the risk of brain tumor. Furthermore, clinical trials exploring the use of FMT to enhance immunotherapy for glioma are also worthy of pursuit.

Previous studies have confirmed that the impact of gut microbiota on human diseases can be driven by its metabolites, including various cancers [20, 54]. For example, gut microbiota can affect the development of lung cancer through the production of short‐chain fatty acids [54, 74]. However, it is still unclear whether the brain tumor‐related gut microbiota identified in this study affects the occurrence of brain tumors through serum metabolites. In this study, we employed mediation MR analysis to investigate the role of 452 serum metabolites as mediators between gut microbiota and brain tumors. However, our analysis did not reveal any evidence supporting the involvement of these serum metabolites as intermediaries. Additionally, we investigated the potential of all 2585 traits from the EBI database to serve as mediators. Notably, employing this approach similar to the phenome‐wide association studies (PheWAS), we discovered that the family *Oxalobacteraceae* may contribute to the tumorigenesis of meningioma by influencing LPC/LPE. LPC is an important inflammatory and nervous system regulator in the human body [75]. There is evidence suggests that LPC possesses the capability to disrupt cell adhesion and trigger apoptosis in liver cancer cells [76]. A report has also confirmed the correlation between LPE and the grade of glioma [77]. Our findings indicate the importance of considering not only broader PheWAS for identifying potential metabolite intermediaries of gut microbiota but also other intermediary factors like immune system alterations. Recent studies have suggested the possibility of direct invasion of tumors by microbes [78, 79], and the microbial peptides derived from the gut microbiota hold promising treatment prospects for brain tumors [80]. Consequently, there is a pressing need for further exploration to elucidate the mechanisms underlying the impact of gut microbiota on brain tumors.

In the reverse MR analysis, we found no genetic evidence supporting a causal effect of brain tumors on gut microbiota. GWAS‐based analysis is unlikely to explain all the genetic heritability of complex traits [81]. TWAS combines gene expression with GWAS to discover complex trait associations at the transcriptome level and increases interpretability. Sherlock, as a TWAS analysis method, integrates all *cis*‐ and *trans*‐eQTL SNPs to determine potential causal relationships between gene expression and traits [43]. In this study, for the first time, the Sherlock comprehensive framework was used to identify genes associated with the abundance of microbiota related to brain tumors in brain tissue. Notably, glioma‐related MRGs demonstrate stronger correlations with the clinical characteristics of tumors compared to MRGs related to benign brain tumors. Consistently, there is a worse dysbiosis in gut microbiota of patients with glioma then those with benign brain tumors [14]. Almost all glioma‐related MRGs are associated with the grade and prognosis of gliomas. As MRGs indirectly reflect the abundance of specific gut microbes, the identified gut microbes not only have causal links with gliomas but may also be involved in the malignant behavior of gliomas. Previous studies have also supported this perspective [70, 79, 82], highlighting the feasibility of improving the prognosis of glioma patients by altering the gut microbiota.

Accurate prognosis assessment holds essential significance in monitoring and guiding personalized treatment strategies and clinical decisions for glioma patients [83]. Presently, there is a scarcity of dependable prognostic biomarkers to identify “high‐risk” glioma patients. Gut microbiota testing presents several advantages, including non‐invasiveness, high diagnostic efficiency, low cost, and accurate diagnosis [84]. However, the development of a gut microbiota‐based prognostic model for glioma remains outstanding, which may require several decades of follow‐up. As an alternative, we developed the MRS model based on 101 machine learning algorithm combinations. MRS not only demonstrates good and stable predictive performance in all public datasets but has also been validated in our in‐house clinical dataset, indicating its excellent generalizability and clinical translational potential. Furthermore, MRS exhibits considerable performance advantages and greater generalizability compared to previously published mRNA signatures. Furthermore, this study found that glioma patients with high MRS have a relatively “hot” TME and higher ICI treatment benefits. Previous studies have shown that immune suppression factors in glioma have a more significant impact on prognosis compared to immune stimulatory factors, and glioma patients with “hot” TME are more likely to respond to ICI treatment [30, 45, 48], potentially due to the activation of pre‐existing anti‐tumor immunity upon improvement of immunosuppression. Since MRS was established based on MRGs and gut microbiota, it not only serves as a robust prognostic and ICI treatment biomarker itself but also provides support for the gut microbiota as prognostic and ICI treatment biomarkers in glioma. Recent studies have also highlighted the significance of the gut microbiota in reshaping the TME of brain tumors and augmenting immune therapy responses [80, 85]. In light of these discoveries and our results, we highly advocate the establishment of clinical cohorts focusing on the gut microbiota in brain tumors, as well as the undertaking of further clinical studies that investigate the potential synergies between the gut microbiota and ICI therapy.

In summary, this study provided genetic evidence supporting the causal impact of gut microbiota on the tumorigenesis of brain tumors, identified MRGs associated with different brain tumors, elucidated the relationship between MRGs and clinical characteristics of brain tumors and established the MRS model for accurate prognosis prediction of patients with glioma and potential insights into the benefits of ICI therapy. Despite these valuable insights, several limitations should be considered. Firstly, the GWAS summary data mainly comprises individuals of European descent, thus, caution must be exercised when generalizing the results to other ethnic groups, and the GWAS data lack stratification by gender or age. Secondly, the sample size involved in the MR‐related analysis is limited, warranting an urgent need for larger sample sizes and more extensive available GWAS summary data to expand the results and explore potential mediators. Thirdly, as an exploratory study, lenient thresholds were employed in the MR analysis, which could increase the likelihood of false positives. Fourthly, the validation of the MRS model in prospective cohorts will further facilitate its clinical translation. Lastly, as mentioned previously, it is extremely necessary to establish brain tumor cohorts that focus on the gut microbiota to determine the vast potential of gut microbes as biomarkers.

## 5 Conclusion

This study confirmed the causal effects of specific gut microbiota on three common brain tumors through various MR analyses, but did not provide evidence supporting the causal effects of brain tumors on gut microbiota. Furthermore, this study identified MRGs that are expressed in brain tissue and correlated with the abundance of gut microbiota. Almost all glioma‐related MRGs are correlated with the grade and prognosis of glioma. The MRS model based on machine learning demonstrated robust predictive efficacy for glioma prognosis and is associated with immunotherapy benefits. The findings of this study expand our understanding of the microbiota‐gut‐brain axis and suggest the establishment of brain tumor cohorts focusing on gut microbiota to further advance the development of gut microbiota‐related biomarkers.

## Author Contributions

Conception and design: Changwu Wu, Jun Tan, and Qing Liu. Collection and generation of data: Changwu Wu, Fushu Luo, Yongye Zhu, and Chunbo Liu. Data analysis and interpretation: Changwu Wu, Fushu Luo, Zheng Chen, and Xiangyu Wang. Manuscript writing and revisions: Changwu Wu, Jun Tan, and Qing Liu. All authors read and approved the final manuscript.

## Ethics Statement

The collection of human tissues was approved by the Medical Ethics Committee of Xiangya Hospital of Central South University (Approval number: 202401003), and written informed consent was provided by all of the patients.

## Conflicts of Interest

The authors declare no conflicts of interest.

## Supporting information



Figures

Tables

## Data Availability

RNA‐seq data (GSA for Human: HRA007149) reported in this study have been deposited in the Genome Sequence Archive in the National Genomics Data Center, China National Center for Bioinformation, Chinese Academy of Sciences, which are publicly accessible at https://ngdc.cncb.ac.cn/gsa. Further information is available from the corresponding author upon reasonable request.
