# Supplementary material for: Deciphering the Potential Causal and Prognostic Relationships Between Gut Microbiota and Brain Tumors: Insights from Genetics Analysis and Machine Learning
Source: Exploration (Beijing). 2025 May 1;5(4):e20240087. doi: 10.1002/EXP.20240087 (PMC12380072; doi:10.1002/EXP.20240087)
Supplement: Supplementary file 1 — Figures [file EXP2-5-e20240087-s001.pdf]

Supplementary Figures

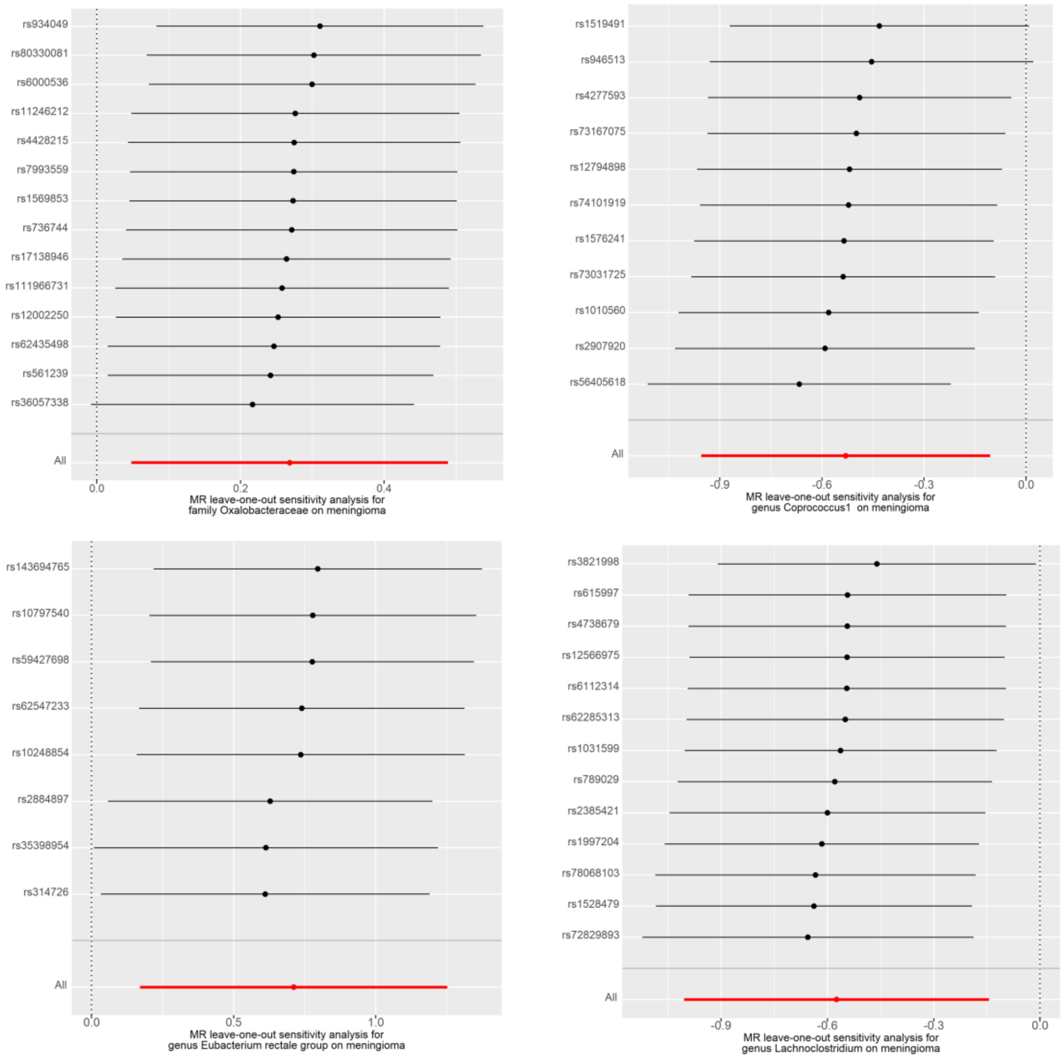

Figure S1. Leave-one-out plots for two sample MR results of causal effects of gut microbes on meningioma. Forest plot of causal estimates omitting each variant in turn.

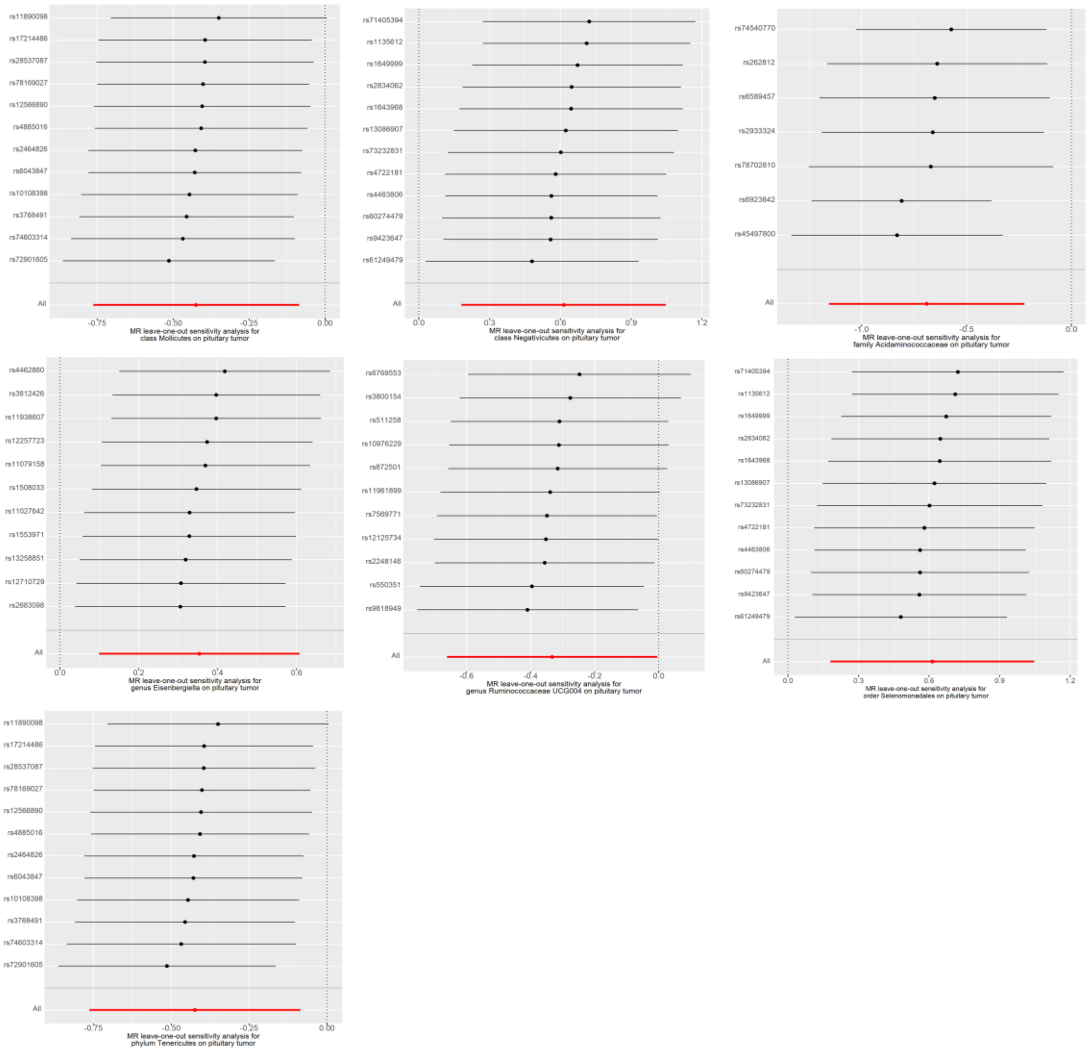

Figure S2. Leave-one-out plots for two sample MR results of causal effects of gut microbes on pituitary tumor. Forest plot of causal estimates omitting each variant in turn.

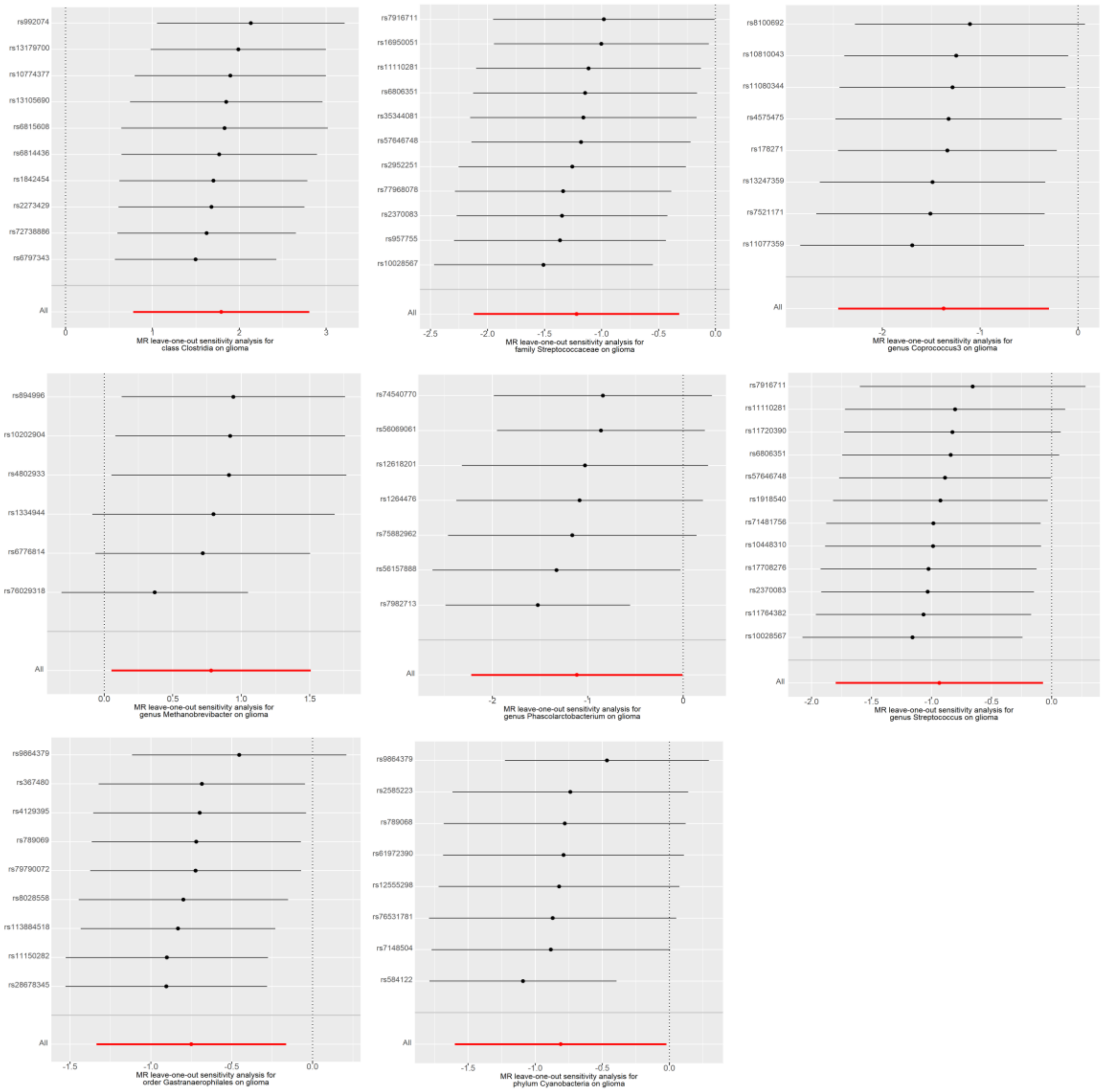

Figure S3. Leave-one-out plots for two sample MR results of causal effects of gut microbes on glioma. Forest plot of causal estimates omitting each variant in turn.

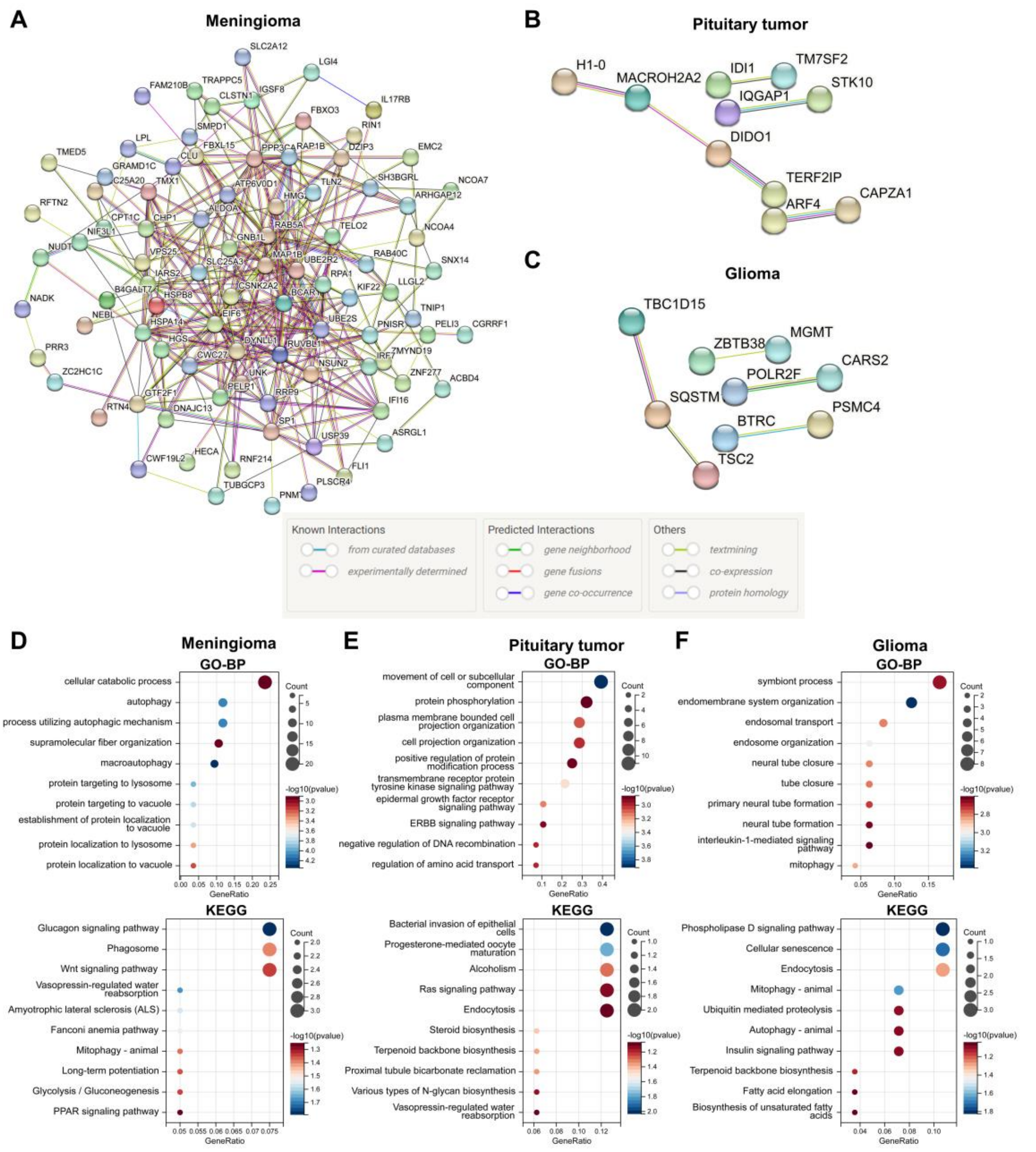

Figure S4. Biological annotation of MRGs. A-C, PPI networks for meningioma-related MRGs (**A**), pituitary tumor-related MRGs (**B**), and glioma-related MRGs (**C**). **D-F**, GO biological processes and KEGG enrichment analysis of meningioma-related MRGs (**D**), pituitary tumor-related MRGs (**E**), and glioma-related MRGs (**F**).

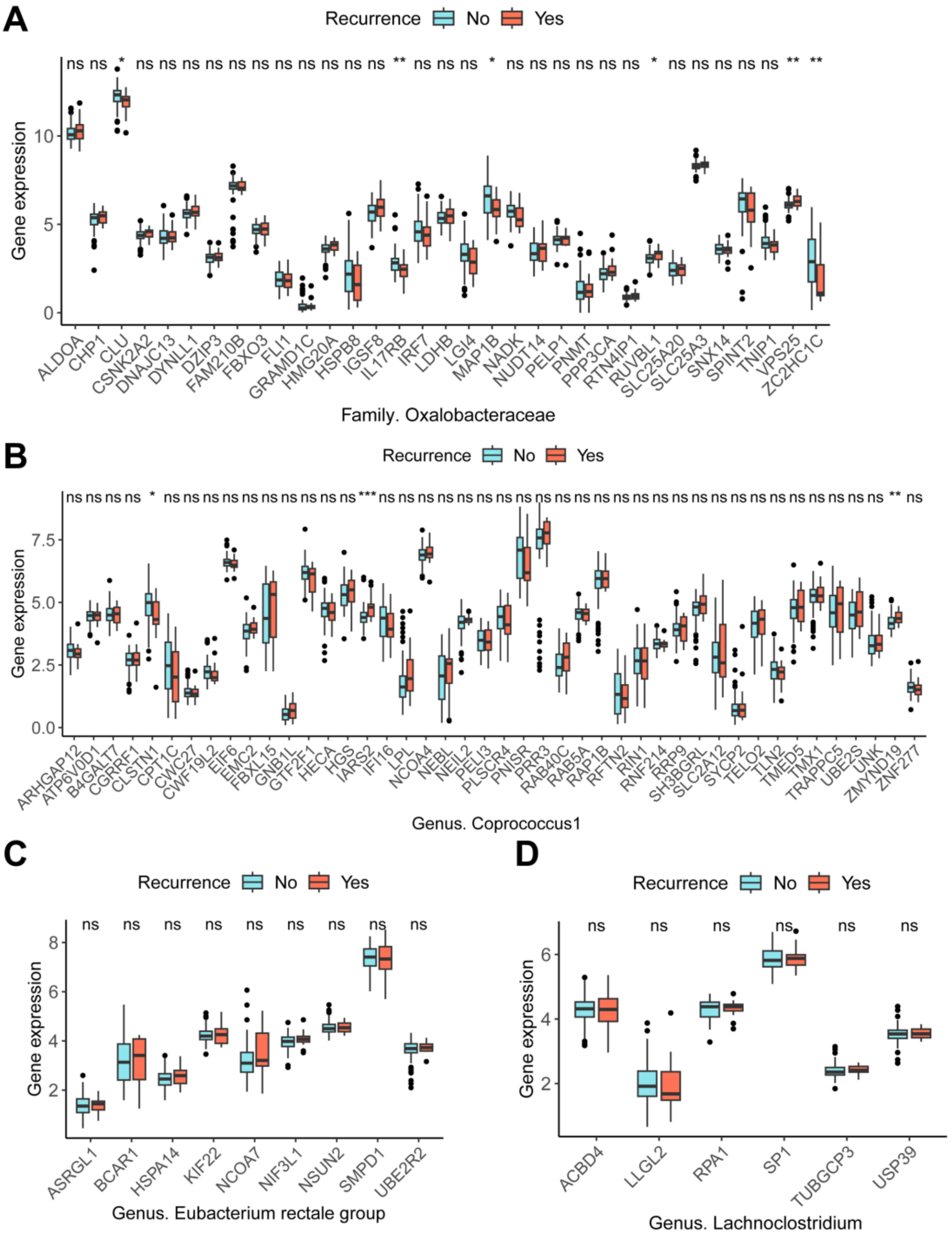

Figure S5. The expression differences of meningioma-related MRGs in meningiomas with and without recurrence. Figures A-D respectively represent the expression of MRGs corresponding to the gut microbe that have a causal effect on meningiomas.

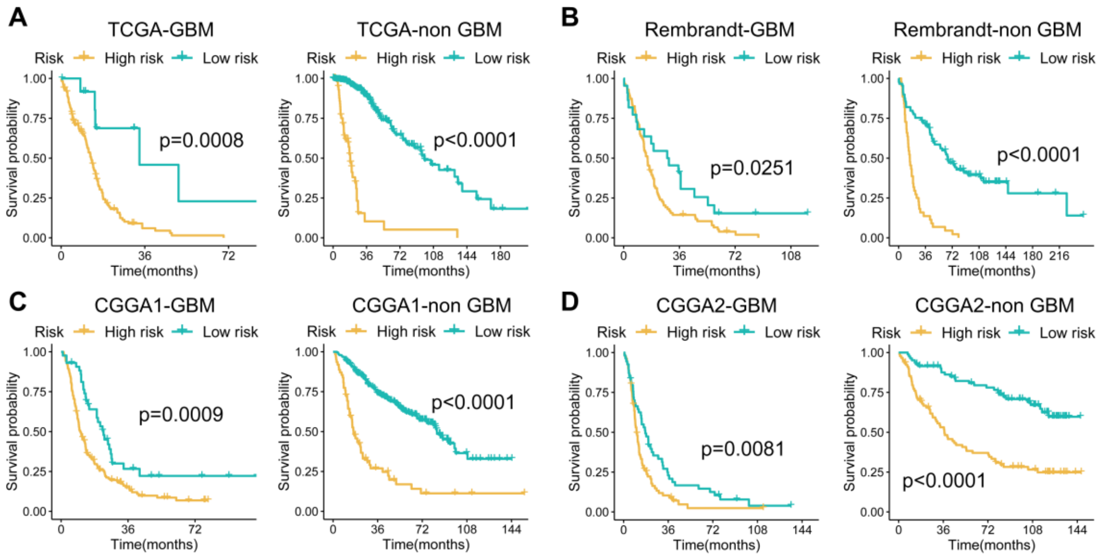

Figure S6. Kaplan-Meier curves depict the OS difference of GBM or non-GBM between MRS-high and MRS-low groups in TCGA (A), Rembrandt (B), CGGA1 (C), and CGGA2 (D) cohorts. Yellow representing the MRS-high group and green representing the pathway MRS-low group.

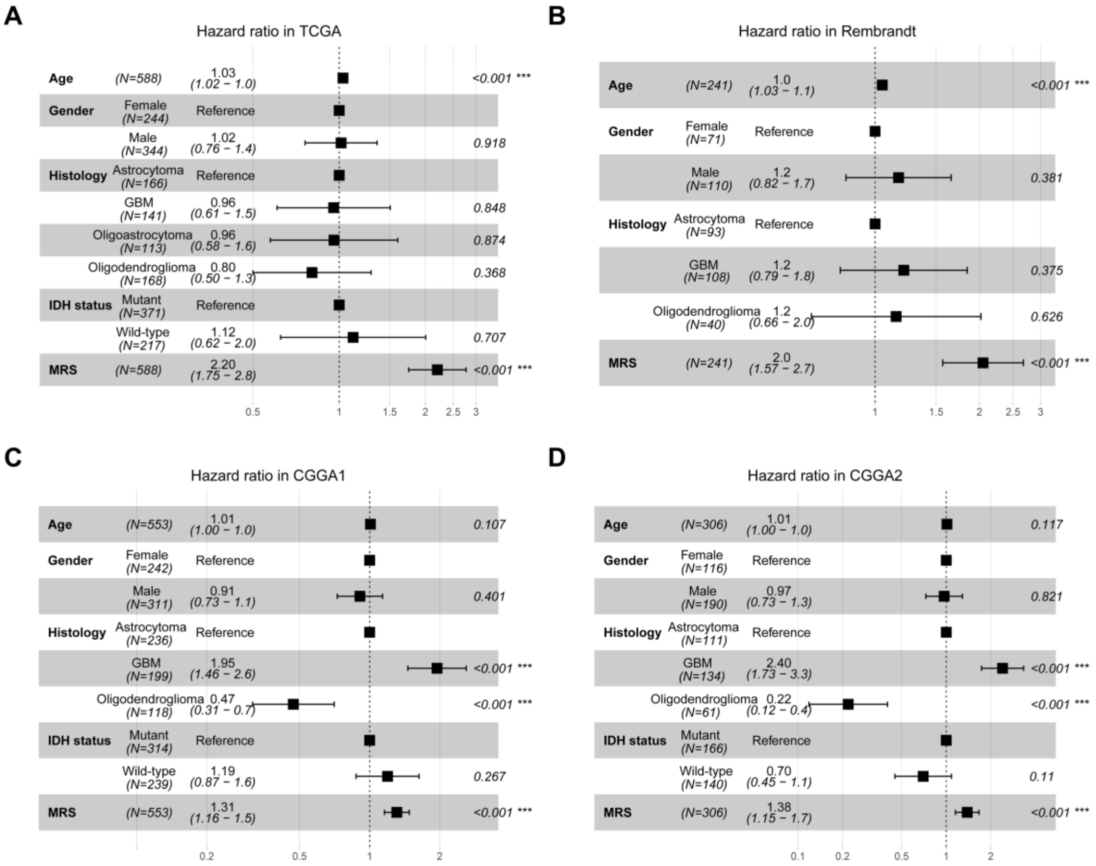

Figure S7. Multivariable Cox regression analysis of OS in TCGA (A), Rembrandt (B), CGGA1 (C), and CGGA2 (D) cohorts. Statistic test: two-sided Wald test. Data are presented as hazard ratio (HR) ± 95% confidence interval (CI).

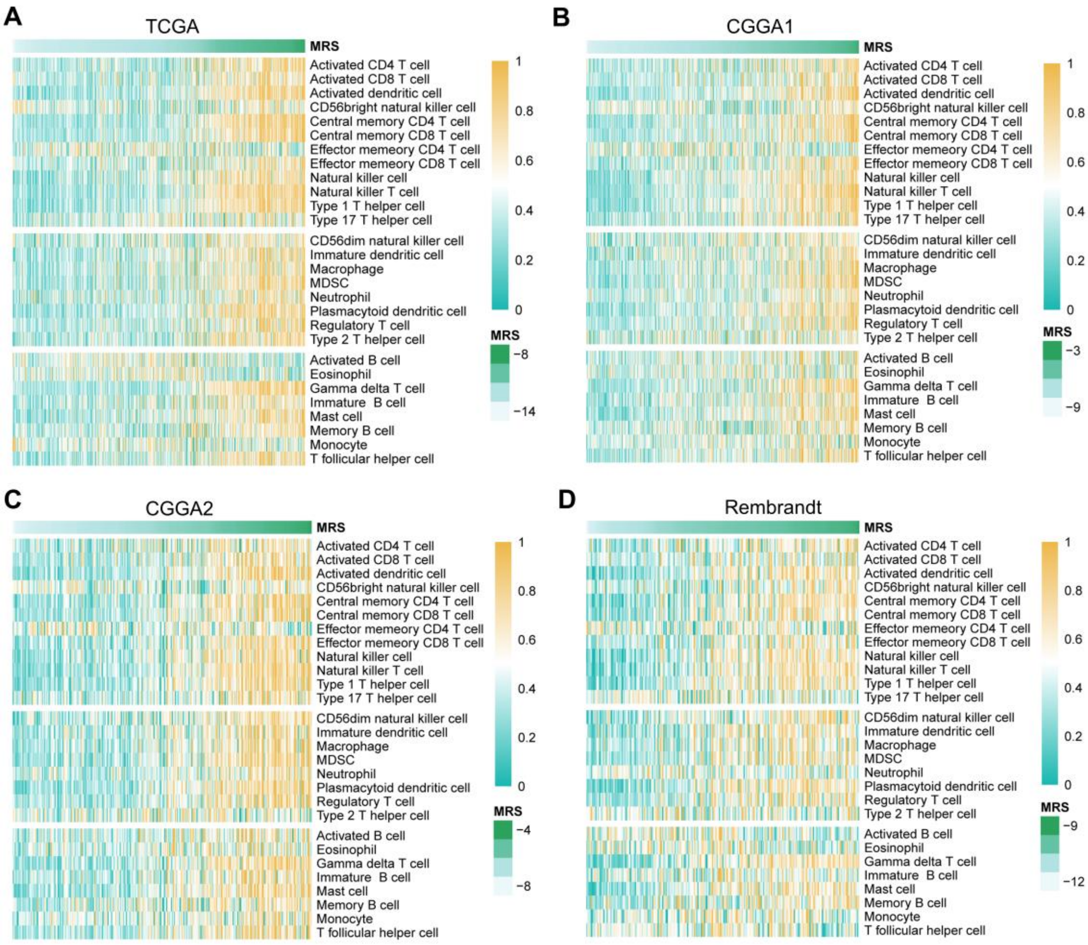

Figure S8. Heatmaps of the relationship between MRS and 28 immune cells in TCGA (A), CGGA1 (B), CGGA2 (C) and Rembrandt (D) cohorts.
